# Letter regarding “Sugammadex vs neostigmine in post-anesthesia recovery: A systematic review and meta-analysis”

**DOI:** 10.17305/bb.2025.13727

**Published:** 2025-12-23

**Authors:** Tuhin Mistry, Abhijit Sukumaran Nair

**Affiliations:** 1 Department of Anaesthesiology and Perioperative Care, Ganga Medical Centre and Hospitals Pvt Ltd, Coimbatore, India; 2 Department of Anaesthesiology, Ibra Hospital, Ibra, Sultanate of Oman

**Keywords:** Sugammadex, neostigmine, neuromuscular block reversal, anesthetic depth, post-anesthesia recovery, airway safety, meta-analysis.

## Abstract

This correspondence comments on the systematic review and meta-analysis by Zhu and Li comparing sugammadex with neostigmine for neuromuscular block reversal and postoperative outcomes. While the authors provide a useful synthesis suggesting faster recovery and less residual blockade with sugammadex, several issues may limit the validity and clinical generalizability of the pooled conclusions. Many key outcomes show extreme heterogeneity (*I*^2^ frequently >90%), raising concerns that combined estimates may obscure clinically important variation in anesthetic technique, blockade depth, monitoring, and recovery protocols. In particular, emergence safety depends not only on neuromuscular indices (e.g., TOF ≥ 0.9) but also on hypnotic depth at the time of reversal; evidence indicates that volatile anesthetic concentration (MAC) can meaningfully modify airway obstruction risk after sugammadex. Additionally, inconsistencies in the reporting of time-based effect sizes, specifically between standardized mean differences (SMD) and mean differences (MD) with identical values, necessitate clarification to enhance interpretability. We highlight the need for more cautious interpretation, targeted subgroup analyses incorporating anesthetic depth and other effect modifiers, and more robust meta-analytic methods to strengthen precision and applicability of the findings.

To the Editor,

We read with great interest the systematic review and meta-analysis by Zhu and Li comparing sugammadex and neostigmine for neuromuscular block reversal and postoperative outcomes [1]. Their synthesis provides valuable insight into an important perioperative issue; however, several methodological and interpretive limitations require clarification to enhance the accuracy and applicability of their conclusions.

First, although the authors report faster recovery and lower residual neuromuscular blockade with sugammadex, these outcomes exhibit significant statistical heterogeneity (*I*^2^ often >90%) [1]. In such cases, pooled effect sizes may obscure clinically meaningful differences related to anesthetic technique, type of surgery, depth of neuromuscular blockade, monitoring methods, and recovery practices. When estimates cluster around the minimal clinically important difference, greater caution is warranted before inferring uniform clinical benefits [2].

Second, recovery was primarily assessed using neuromuscular indices such as TOF ≥ 0.9 and extubation time; however, clinical emergence physiology depends equally on the depth of hypnosis at the time of reversal, which influences airway safety during emergence. Kang et al. demonstrated that synchronizing the timing of consciousness recovery with neuromuscular recovery is essential for safe emergence. Isolated restoration of muscle tone while hypnotic depth persists increases the risk of airway obstruction [3]. Their study found that administering sugammadex at ≥ 0.3 minimum alveolar concentration (MAC) of volatile anesthetic doubled the risk of airway obstruction, while administration at <0.3 MAC was significantly safer. These clinically relevant modifiers, such as MAC at reversal, inhalational agent, age, and duration of surgery, influence emergence time and airway safety but were not explored in subgroup analyses. Omission of these modifiers likely drives the high heterogeneity (*I*^2^ > 90%) and limits generalizability; therefore, integrating them into subgroup frameworks is necessary for accurate interpretation.

Third, the reporting of effect sizes requires clarification. Time-based outcomes were presented as standardized mean differences (SMD) in the figures but as mean differences (MD) with identical numerical values in the tables, creating an important inconsistency. If SMDs were utilized, such unusually large magnitudes necessitate contextualization; if MDs were intended, units should be specified. Accurate reporting of effect sizes is essential for clinicians to determine whether statistically significant differences translate into meaningful clinical improvements.

Fourth, given the extreme heterogeneity and variable event rates, more robust meta-analytic approaches, such as generalized linear mixed models, variance-stabilizing transformations, or trial sequential analysis, would enhance reliability [4]. Recent perioperative evidence indicates that sugammadex does not uniformly reduce postoperative pulmonary complications, particularly when confounding factors and risk adjustments are considered [5, 6]. Contextualizing pooled results within these findings is crucial.

In conclusion, Zhu and Li provide a valuable synthesis supporting the pharmacological efficacy of sugammadex; however, anesthetic depth at reversal, airway physiology, and recovery-modifying factors remain unaddressed. Incorporating these variables and employing more rigorous analytic safeguards would enhance the precision and clinical relevance of their conclusions.
